# Expanding the phenotypic spectrum of *FGF12*-epilepsy—does prompt precision therapy affect outcomes?

**DOI:** 10.1038/s41525-026-00581-0

**Published:** 2026-05-25

**Authors:** Leo Arkush, Kristina Karandasheva, Frédérique Ouellet, Alissa M. D’Gama, Beth Rosen Sheidley, Nicole S. Y. Liang, Vann Chau, Gregory Costain, Lacey Smith, Anoushka Alwis, Christin Eltze, Annapurna Poduri, Felice D’Arco, Josh Adams, Kristen Lee, Jaspal Singh, Alexander P. Y. Brown, Arjuna Nagendran, Ben Pode-Shakked, Michal Tzadok, Bruria Ben Zeev, Amy McTague

**Affiliations:** 1 Pediatric Neurology Unit, Edmond and Lily Safra Children’s Hospital, Sheba Medical Center, Ramat Gan, Israel; 2 Gray Faculty of Medical and Health Sciences, Tel Aviv University, Tel Aviv, Israel; 3 Institute of Neuropathology, Universitätsklinikum Erlangen, Erlangen, Germany; 4 Division of Neurology, The Hospital for Sick Children (SickKids), Toronto, ON Canada; 5 Department of Paediatrics, University of Toronto, Toronto, ON Canada; 6 Epilepsy Genetics Program, Boston Children’s Hospital, Boston, MA USA; 7 Division of Newborn Medicine, Department of Pediatrics, Boston Children’s Hospital, Harvard Medical School, Boston, MA USA; 8 Division of Clinical and Metabolic Genetics, The Hospital for Sick Children (SickKids), Toronto, ON Canada; 9 Program in Genetics & Genome Biology, SickKids Research Institute, Toronto, ON Canada; 10 Department of Neurology, Boston Children’s Hospital, Boston, MA USA; 11 Neurology, Great Ormond Hospital for Children, London, UK; 12 Neuroradiology, Great Ormond Hospital for Children, London, UK; 13 Division of Pediatric Neurology, University of Michigan, Ann Arbor, MI USA; 14 Division of Pediatric Genetics, Metabolism and Genomic Medicine, Ann Arbor, MI USA; 15 Department of Paediatric Neurology, University Hospital Southampton, Southampton, UK; 16 Infection, Immunity and Inflammation, UCL Great Ormond Street Institute of Child Health, London, UK; 17 Department of Clinical Neurophysiology, National Hospital for Neurology and Neurosurgery, London, UK; 18 The Institute for Rare Diseases, Edmond and Lily Safra Children’s Hospital, Sheba Medical Center, Ramat Gan, Israel; 19 Developmental Neurosciences, UCL Great Ormond Street Institute of Child Health, London, UK

**Keywords:** Diseases, Genetics, Medical research, Neurology, Neuroscience

## Abstract

Fibroblast growth factor-12 (*FGF12*) variants have been associated with developmental and epileptic encephalopathy (DEE) with evidence of modulation of voltage-gated sodium channels Na_v_1.2 and Na_v_1.6. We aim to expand the phenotypic spectrum of *FGF12-*related epilepsy with emphasis on precision therapy. We describe 12 patients: eight with neonatal onset seizures with the recurrent p.Arg52His (c.155 G > A) variant, two with a previously unreported p.Glu153Gly (c.458 A > G) variant, one with a p.Gly50Ser (c.148 G > A) variant, and one with a de novo whole-gene duplication. Atypical absence seizures were present in 5/12 patients. Brain MRI was normal in 10/12; one patient’s MRIs showed progressive cerebellar atrophy, and one patient’s MRI showed a hemispheric infarct. 8 patients promptly started on sodium channel blockers became seizure-free with good developmental outcomes while 4 developed DEE. In summary, we expand the phenotypic spectrum of *FGF12-*related epilepsy and discuss the role of early precision therapy in developmental and epilepsy outcomes.

## Introduction

Epilepsy affects up to 0.7% of children, with seizures often significantly impacting quality of life^1^. Children presenting with epilepsy in the neonatal and infant age group have a high burden of morbidity, drug resistance, and global developmental delay^2^. Etiology can be identified in approximately half of children presenting with early-onset epilepsy, and most genetic causes reported to date are de novo monoallelic variants^2^. Alongside increasing yield of genetic diagnostic testing^3^, evidence of efficacy of precision therapies in childhood epilepsy remains largely limited to genetic metabolic disorders, epilepsies related to mTOR pathway dysfunction, and some channelopathies^4^. There remains substantial unmet need for effective, well-tolerated precision therapies for pediatric epilepsy^5^.

Variants in Fibroblast growth factor-12 (*FGF12*, MIM #601513), previously known as *FHF1*, have been associated with developmental and epileptic encephalopathy (DEE) with neonatal onset (DEE47, MIM #617166). FGF12 is involved in signaling cascades via FGF receptors to prevent apoptosis, ribosomal biogenesis and cytoskeletal regulation but the main role is modulation of voltage-gated sodium channels^6^. There is recent evidence that *FGF12* pathogenic variants modulate Na_v_1.2 and Na_v_1.6, with complex, variant-type dependent gating changes resulting in epilepsy and neurodevelopmental disease^7^. FGF12 also interacts with Na_v_1.5^8^ and Na_v_1.9^9^.

Previously report cohorts have revealed two recurrent *FGF12* variants that accounted for the vast majority of cases: p.Arg52His (c.155 G > A) and p.Gly50Ser (c.148 G > A) according to the NM_004113.6 transcript (previously FHF1 p.Arg114His, c.341 G > A, and p.Gly112Ser c.334 G > A, NM_021032.4 transcript). A series of 17 cases, of whom 14 had the recurrent p.Arg52His (c.155 G > A) variant, reported poor seizure and developmental outcomes in most patients^10^. They note that phenytoin was effective in 35% of patients but the time interval between seizure onset and start of sodium channel blocker (SCB) therapy is not reported. Pierret and colleagues describe 10 additional patients with the p.Gly50Ser or p.Arg52His recurrent variants who developed epilepsy before 5 months, with 70% achieving excellent seizure control and good developmental outcomes^11^. They did not report a correlation between early sodium channel blocker therapy and neurodevelopmental outcomes. An additional report describes good seizure control in patients with the recurrent p.Arg52His variant with sodium valproate and topiramate^12^.

9 patients have also been described with partial duplications of the *FGF12* gene, who tended to present later, with variable outcomes; some responded well to sodium channel blockers while others developed drug-resistant epilepsy with developmental regression^13–16^. Unlike most variants with single nucleotide changes, some duplications caused aberrant, often longer transcripts, which are thought to lead to non-functional protein and nonsense mediated decay^16^.

We report an international retrospective series of 12 unpublished patients with *FGF12*-epilepsy, including those with early diagnosis and prompt initiation of SCBs, present in-silico modeling and summarize the FGF12 landscape.

## Results

### Epilepsy phenotype of previously unreported patients

**Fig. 1:**
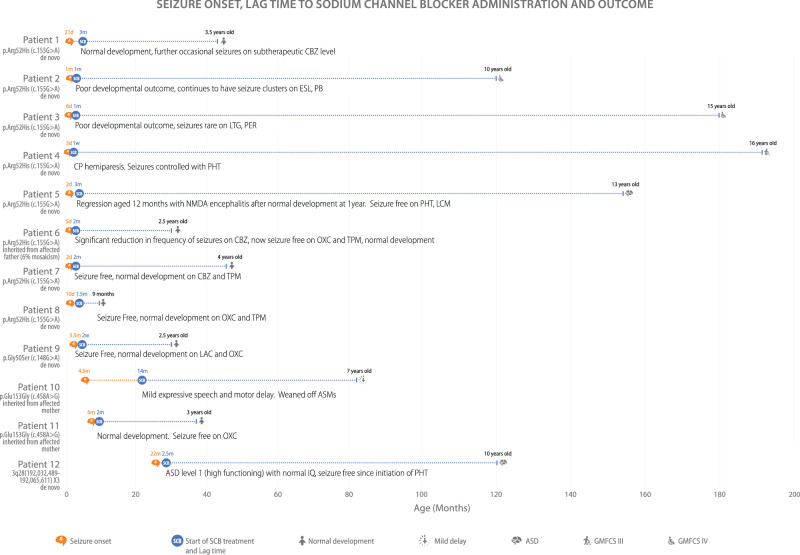
Timeline demonstrating the clinical course of patients described. ASD = autism spectrum disorder. CBZ = carbamazepine. ESL = eslicarbazepine. GMFCS = Gross Motor Function Classification System. LTG = lamotrigine. LCM = lacosamide. OXC = oxcarbazepine. PB = phenobarbitone. PER = perampanel. PHT = phenytoin. SCB = sodium channel blocker. TPM = topiramate.

We describe 11 patients with variants in *FGF12* and one with a partial chromosomal duplication. Patients’ clinical, EEG, neuroradiological and genetic details are summarized in Table 1. 10 patients are unrelated, while 2 are siblings. 6 patients are female. The median age at time of enrollment was 5.5 years (range 9 months to 16 years, standard deviation 5.1 years). Fig. 1 graphically presents age of epilepsy onset and outcomes, antiseizure medicines (ASMs), and developmental/intellectual trajectories. Patient 1 is a 3-and-a-half year-old female of white descent born to healthy, unrelated parents at term. She presented aged 3 weeks of age with seizures described as eyelid flickering, eye deviation, pauses in breathing, and tonic-clonic activity. Seizures continued despite levetiracetam and phenobarbital treatment until transition to carbamazepine at 4 months of age, which was followed by seizure freedom. She had an episode of status epilepticus aged 10 months associated with subtherapeutic levels of carbamazepine. She has since had occasional seizures when carbamazepine levels drop but is well controlled on 35 mg/kg/day. She has achieved all developmental milestones on time.

**Table 1:** Phenotype and genotype data for newly reported patients with *FGF12*-epilepsy

PT ID	Gender/Age at last observation	FGF12 variant, inheritance	Epilepsy onset	Seizure type	Epilepsy Type	SE (frequency)	Interictal EEG	Ictal EEG	ASM	ASM efficacy	ID	ASD/other	First brain MR/age	Second brain MR/age	Lag between seizure onset and SCB
1	F/3.5Y	p.Arg52His (c.155 G > A) de novo	21 d	GTCS, A, eye deviation	Combined generalized and focal epilepsy	No	Transient posterior sharp waves	Not performed	PB, LEV, CBZ	CBZ	No	No	Normal	No	3 m
2	F/10Y	p.Arg52His (c.155 G > A) de novo	1 m	GTCS, A	Combined generalized and focal epilepsy	frequent	Periodic burst suppression	Ictal activity migrating from left to right aborted by lidocaine infusion	CLB, CBZ, LEV, PB, mexiletine, OXC, LTG, ESL	Partially responsive to LTG, ESL. Acute response to mexiletine, lidocaine	Severe	Hemiplegia secondary to infarct	Nonspecific white matter right parietal edema, likely secondary to seizures/1 m	Infarct in the left cerebral hemisphere with associated atrophic/gliotic changes/3 yr	1 m
3	F/15Y	p.Arg52His (c.155 G > A) de novo	6 d	GTCS, A	Combined generalized and focal epilepsy	No	Focal changes	Not performed	LTG, PER, TPM, PHT, KD	LTG, PHT,TPM, PER	Severe	Limb hypotonia, CP GMFCS IV	Normal/9 d	No	1 m
4	F/16Y	p.Arg52His (c.155 G > A) de novo	3 d	GTCS, A	Combined generalized and focal epilepsy	Frequent	Normal	Not performed	STI, PB, PHT, LEV, CLB, CZP, CBZ, TPM, LTG, VPA, RFM	PHT, LAC	Severe	Limb hypertonia, right hemiparesis, incoordination	Normal/1 m	Progressive cerebellar atrophy with sparing of pons/4 y	1w
5	M/13Y	p.Arg52His (c.155 G > A) de novo	2 d	MS, CS	Combined generalized and focal epilepsy	No	Multi focal slow generalized SW outbursts with suppression	Not performed	VPA, LEV, TPM, LTG, CZP, CLB, SLT, CBZ, OXC, CBD, ZNS, PHT, LAC	PHT, LAC	Severe	ASD level 3	Normal/18 m	Normal/2 yr	3 m
6	M/2.5Y	p.Arg52His (c.155 G > A) inherited from affected father (6% mosaicism)	5 d	TS	Generalized	No	Intermittent sharp waves in bilateral posterior regions	Not performed	PB, LEV, CBZ	CBZ, TPM	No	No	Normal/1 m	No	2 m
7	F/4Y	p.Arg52His (c.155 G > A) de novo	2 d	Apneas, TS, CS	Generalized	No	Normal	Not performed	PB, TPM, CBZ	CBZ, TPM	No	No	Normal/1 m	No	2 m
8	M/9 M	p.Arg52His (c.155 G > A) de novo	10 d	FIAS	Focal	2 events aged 3 m and 4 m	Independent bilateral temporal spike and wave discharges	Not performed	OXC, TPM	OXC, TPM	No	No	Normal/2 m	No	1.5 m
9	M/2.5Y	p.Gly50Ser (c.148 G > A) de novo	3.5 m	GTCS	Focal	No	Occasional independent left and right temporal sharp waves	Temporal (focal)	LEV, OXC, LAC	OXC, LAC	No	No	Normal/4 m	No	2w
10	F/7Y	p.Glu153Gly (c.458 A > G) inherited from affected mother	4.5 m	Behavioral arrest, cyanosis	Focal epilepsy	No	Normal	Left temporal (focal) onset	PB, LEV, OXC	OXC	Mild	No	Normal/6 m	No	14 m
11	M/3Y	p.Glu153Gly (c.458 A > G) inherited from affected mother	6 m	TS	Focal epilepsy	No	Rare bilat. frontal and right posterior temporal sharp waves in sleep	Not performed	LEV	OXC	No	No	Normal/6 m	No	2 m
12	M/10Y	3q28(192,032,489-192,065,611) X3 de novo	22 m	A	Combined generalized and focal epilepsy	No	Generalized and multifocal spike wave discharges	Not performed	VPA, CZP, LEV, TPM, PHT	PHT, OXC	No	ASD level 1	Normal/22 m	Normal/3 y	2.5 m

Seizure type abbreviations: *A* absence seizures, *Apneas* apnea-related events, *Behavioral arrest* behavioral arrest episodes, *CS* clonic seizures, cyanosis cyanotic episodes associated with seizures, *eye deviation* epileptic eye-deviation events, *FIAS* focal impaired-awareness seizures, *GTCS* generalized tonic–clonic seizures, *MS* myoclonic seizures, *TS* tonic seizures.

Antiseizure medicines abbreviations: *CBD* cannabidiol, *CBZ* carbamazepine, *CLB* clobazam, *CZP* clonazepam, *ESL* eslicarbazepine acetate, *KD* ketogenic diet, *LAC* lacosamide, *LEV* levetiracetam, *LTG* lamotrigine, *mexiletine* mexiletine, *OXC* oxcarbazepine, *PB* phenobarbitone, *PER* perampanel, *PHT* phenytoin, *RFM* rufinamide, *SLT* sulthiame, *STI* stiripentol, *TPM* topiramate, *VPA* valproate, *ZNS* zonisamide.

Patient 2 is a 10-year-old female born to healthy unrelated parents of White/South Asian background who presented at age 4 weeks, following a normal perinatal and newborn course, with tonic seizures and status epilepticus that responded to phenytoin and levetiracetam. At the age of 4 months she had super-refractory status epilepticus, which only abated after IV lidocaine; she was then transitioned to mexiletine. She subsequently received clobazam, carbamazepine, and oxcarbazepine, which was stopped due to hyponatremia; she is currently treated with lamotrigine, eslicarbazepine and phenobarbital and has clusters of generalized tonic-clonic seizures every 1-2 weeks. She previously had frequent episodes of behavioral arrest. She has significant hypotonia, right hemidystonia, cerebral palsy GMFCS Level IV and smiles responsively with no speech development.

Patient 3 is a 15-year-old girl born to unrelated parents who presented with focal tonic seizures aged 6 days, with partial responsiveness to phenytoin. Seizure control later improved on the ketogenic diet, phenytoin and, topiramate. She is currently treated with lamotrigine and perampanel and has infrequent tonic-clonic seizures (1/year) and occasional behavioral arrest events. She is hypotonic with diminished deep tendon reflexes and non-ambulant consistent with cerebral palsy GMFCS Level IV.

Patient 4 is a 16-year-old girl born after an unremarkable pregnancy with no family history who presented with focal and bilateral motor seizures aged 3 days, with recurrent status epilepticus and atypical absences. Seizures were responsive to phenytoin and recurrent seizures were associated with low serum levels. She has developmental delay, limb hypertonia, right hemiparesis and incoordination.

Patient 5 is a 13-year old boy born at term to parents of Sephardic (Libyan) Jewish background. He presented with seizures aged 2 days of life resistant to drug treatment until initiation of oxcarbazepine therapy aged 3 months. He was then seizure-free until the age of 1 year and achieved all normal developmental milestones at this age. In his second year of life, he had a significant regression in terms of cognitive and motor milestones with return of seizures associated with NMDA encephalitis (diagnosed by CSF antibody elevated anti-NMDA titers). He is now ambulant, non-verbal with severe intellectual disability (ID), autism spectrum disorder (ASD), and a recent diagnosis of Crohn’s disease. He was previously diagnosed with Lennox-Gastaut syndrome according to seizure and EEG features, and received sodium valproate, levetiracetam, topiramate, lamotrigine, clonazepam, clobazam, sulthiame, carbamazepine, oxcarbazepine, cannabidiol and zonisamide. He is currently seizure-free on phenytoin and lacosamide.

Patient 6 is a 2-and-half year old male born to Caucasian parents with an unremarkable course who presented aged 5 days with frequent seizures characterized by tonic posturing and lip smacking. Initially treated with levetiracetam and phenobarbital, he had significant reduction of seizure frequency to less than 1/month on addition of carbamazepine (with subtherapeutic levels) aged 2 months with weaning of other ASMs. He was later transitioned to oxcarbazepine and topiramate, achieving seizure freedom. Developmental milestones have been achieved with no delay. His father has a background of 4 previous seizures following mild head injuries or sleep deprivation and is well controlled on carbamazepine.

Patient 7 is a 4-year old female born at term of Chinese origin who presented with apneas, and both tonic and clonic seizures aged 2 days. She did not respond to treatment with phenobarbital but responded to topiramate and later to carbamazepine commenced at 2 months of age. She has been seizure free since then. She has attained all her developmental milestones.

Patient 8 is a 9-month old boy with episodes from day 10 of age that were initially presumed to be related to gastroesophageal reflux disease. At 2 months of age, he presented with focal impaired awareness seizures associated with gaze fixation to the left and facial twitching, and non-responsiveness. He responded partially to oxcarbazepine but returned at age 3 and 4 months with status epilepticus events, and has since been seizure-free following addition of topiramate. Development is normal for age.

Patient 9 is a 2-and-half-year old male who presented with normal early development aged 3.5 months with a febrile seizure provoked by a urinary tract infection. Levetiracetam was not effective with recurrent seizures while afebrile, while he responded to lacosamide. Due to breakthrough seizures aged 5.5 months, oxcarbazepine was added, and he has since been seizure free for 2 years on dual SCB therapy. He has attained all developmental milestones on time.

Patient 10 is a 7-year old female with a normal course prior to seizure onset aged 4.5 months. Seizures involved behavioral arrest and cyanosis and were not responsive to phenobarbital or levetiracetam, but she has been seizure-free since oxcarbazepine was commenced aged 19 months. She was weaned off ASM therapy at age 4 and has mild expressive speech and motor delay. Her brother, Patient 11, started having asymmetric tonic seizures with mild perioral cyanosis and cardiorespiratory compromise at age 6 months, which did not respond to levetiracetam but resolved aged 8 months on oxcarbazepine. He is now 3 years old with normal development. Their mother had generalized seizures from 6 months of age, treated with phenobarbital, with resolution at 2 years.

Patient 12 is a 10-year old boy born at term to unrelated Ashkenazi Jewish parents presenting with focal behavioral arrest seizures aged 22 months. Seizures were refractory to treatment with sodium valproate, levetiracetam, clobazam, topiramate, clonazepam, and the ketogenic diet until phenytoin was commenced 2.5 months following seizure onset. Since then, he has been seizure-free on low-dose phenytoin monotherapy following weaning of other ASMs. He was recently transitioned to oxcarbazepine to prevent potential adverse affects from long-term phenytoin administration. Prior to seizure onset, he had mild developmental delay. He regressed with seizure onset, with full return to baseline following phenytoin initiation. He was later diagnosed with ASD Level 1, but has normal IQ (95) and educational attainments.

### Response to sodium channel blockers and other antiseizure medicines

All twelve patients exhibited responsiveness to at least one SCB, including oxcarbazepine in five patients and phenytoin in four, while carbamazepine, lamotrigine, and lacosamide were each effective in three patients. During the acute status epilepticus phase, one patient responded to intravenous lidocaine and was subsequently maintained on mexiletine. Additional benefit was observed with topiramate in four patients and perampanel in one. Prior to initiation of SCB therapy, all patients had failed to respond to first- and second-line antiseizure medications, most of whom presented in the neonatal period or early infancy and had been treated primarily with phenobarbitone and levetiracetam. No clinical improvement was documented in those who had previously received sodium valproate or benzodiazepines, including clobazam or clonazepam.

### Neurophysiological assessment and cardiac abnormalities

**Fig. 2:**
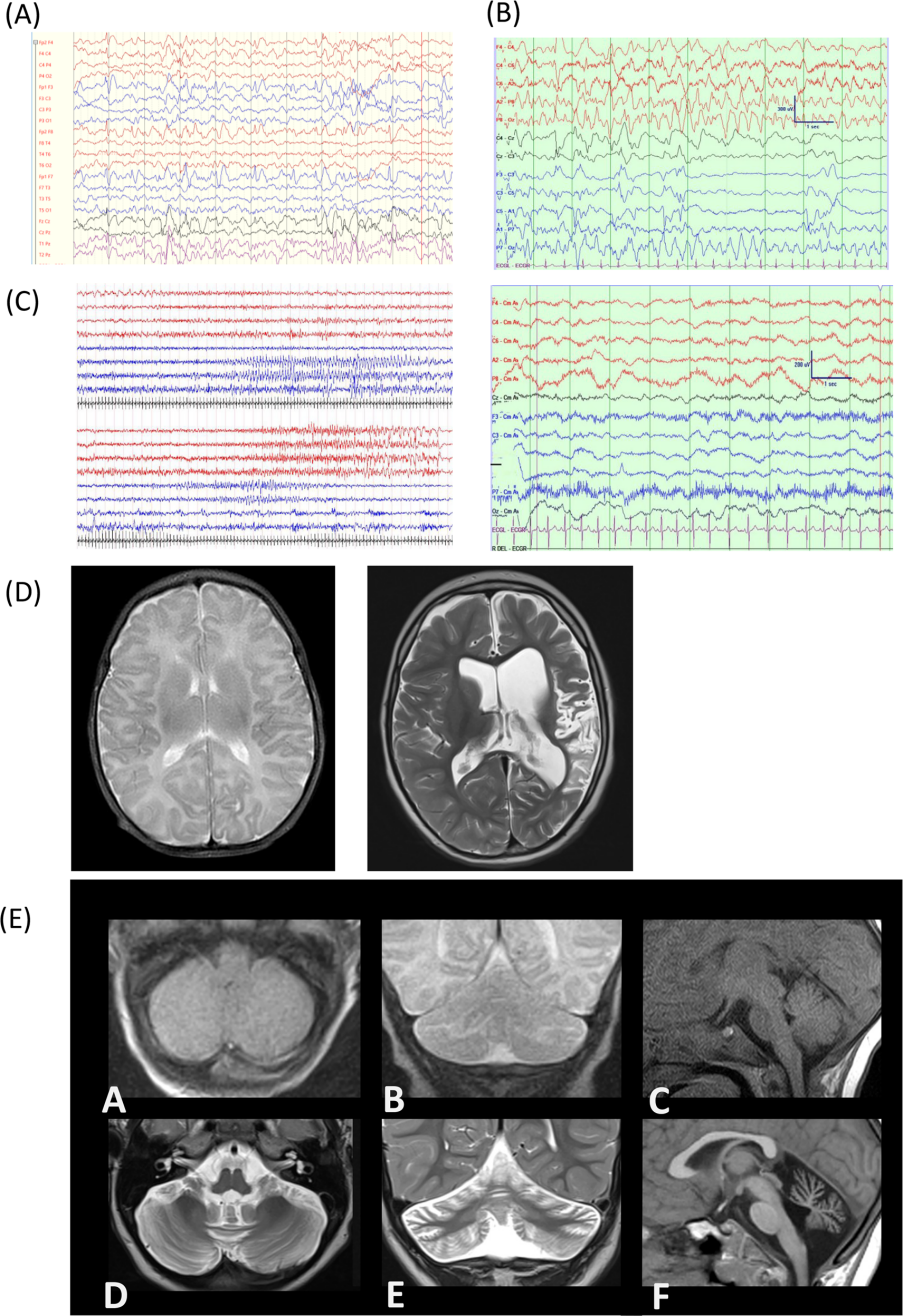
Electroencephalographic and neuroradiological findings. **A** Patient 12 – Interictal EEG demonstrating bilateral frontal spike-wave epileptiform activity, more prominent on the left. **B** Patient 3 – Ictal EEG demonstrating emergent amplitude asymmetry (R > L) evolving to asymmetrical left sided decrement associated with asynchronous clonic jerking. **C** Patient 2 – The left panel shows ictal EEG aged 4 months prior to lidocaine infusion. The EEG shows very frequent seizures in the left hemisphere (blue) spreading to the right hemisphere (red). The right panel shows EEG on intravenous lidocaine, capturing stiffening episodes with no EEG correlate. Epileptiform activity has disappeared. The background is slow as anticipated with sedation. **D** Patient 2 - Left hemisphere infarct. Axial MRI brain images show normal appearances aged 4 weeks (left) and evolution of a mature left hemisphere infarct aged 3 years (right). This is suspected the sequelae of super-refractory status epilepticus in the first year of life. **E** Patient 4 - Coronal and axial T2 weighted-images (**A** and **B**) and sagittal T1 weighted-images (**C**) when the patient was 1-month-old showing normal appearances of the posterior fossa structures. Follow-up magnetic resonance scan: coronal and axial T2 weighted-images (**A** and **B**) and sagittal T1 weighted-images (**C**) when the patient was 4 year-old showing development of global cerebellar atrophy without involvement of the pons and with normal cerebrum (“pure cerebellar atrophy”).

Interictal electroencephalography (EEG) demonstrated non-specific focal or multi-focal and generalized spikes, or spike-wave activity in most patients (for example in Patient 12, with bilateral frontal spike-wave epileptiform activity, more prominent on the left in Fig. 2a), but we note that posterior and temporal spikes or spike-and-wave activity were predominant (in 7/12 patients). Burst suppression was seen in one case [Patient 2], with three patients having a normal interictal EEG [Patients 4, 7, 10]. No patients developed hypsarrhythmia. Ictal EEGs demonstrated focal onset (Fig. 2b). Fig. 2c demonstrates a migrating seizure pattern seen in Patient 2 at age 4 months with seizure cessation on lidocaine injection. Most patients had normal interictal EEGs since seizure resolution. All patients with documented cardiology evaluations had no reports of cardiac abnormalities.

### Neuroimaging

Brain magnetic resonance imaging (MRI) was normal in 10/12 patients. Patient 2 had a normal MRI aged 4 weeks with later evolution of left hemisphere infarct thought to be a sequela of super-refractory status epilepticus in infancy (Fig. 2d). MRI of Patient 4 aged 4 years demonstrated progressive cerebellar atrophy with sparing of pons (Fig. 2e) which may be associated with prolonged phenytoin administration.

### Epilepsy syndromes

Of patients with a specific epilepsy syndrome, 3 (Patients 2, 3 and 4) had early infantile developmental and epileptic encephalopathy, and 2 (Patients 10 and 11) had self-limited familial neonatal-infantile epilepsy. 7 of the patients have neonatal-infantile onset epilepsy generally responsive to SCB therapy with normal developmental outcomes in 6, not consistent with a particular epilepsy syndrome^17^.

### Genetic testing

1 diagnosis was made on chromosomal microarray, 5 on genome sequencing (including 2 via rapid genome sequencing in the Gene-STEPS study)^3^, 5 on exome sequencing and one on Next Generation Sequencing (NGS)-based gene panel sequencing. 8 were found to have the identical recurrent variant p.Arg52His (c.155 G > A), 7 of which were de novo and inherited in one case (his mildly affected father had 6% mosaicism). Patient 9 has a previously reported recurrent p.Gly50Ser (c.148 G > A) de novo variant. Patients 10 and 11 had a novel maternally inherited variant p.Glu153Gly (c.458 A > G), with a second de novo potential contributary variant in *STAG1* in Patient 11^18^, but following identification of the *FGF12* variant in Patient 11 and her affected mother, this was not thought to be significant. Patient 12 has a novel de novo intragenic gain encompassing exon 4 of *FGF12* (GRCh38:chr3:192,314,700-192,347,822×3).

### In-silico modeling

**Fig. 3:**
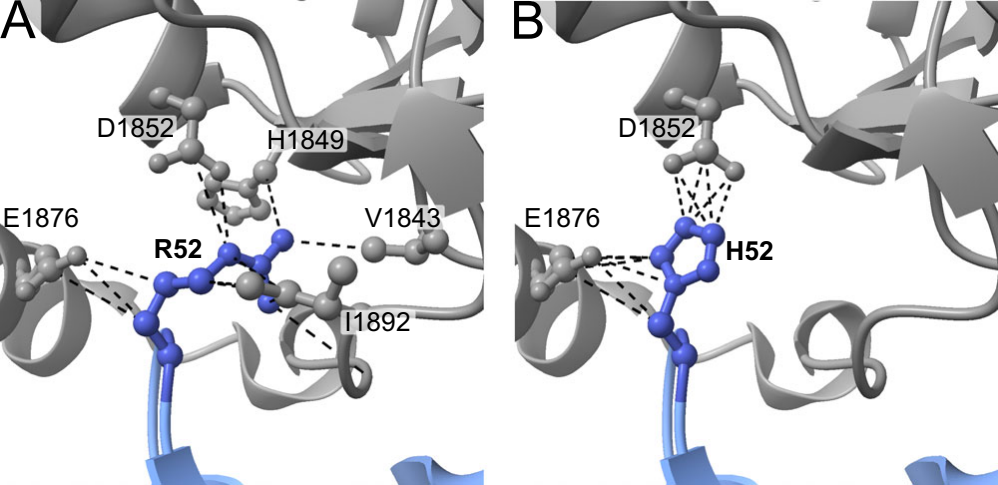
Structural modelling predicts altered local interactions at the FGF12–Nav1.5 interface following the R52H substitution. **A** Wild-type and (**B**) R52H models of the FGF12-Nav1.5 interaction interface. The structural model was generated from the homologous FGF13-Nav1.5-calmodulin complex (PDB: 4DCK; 2.20 Å)^31^, with residue numbering mapped to the corresponding FGF12 sequence. Arg52 is located within the FGF12-Nav1.5 interaction interface. Substitution of Arg52 by His is predicted to alter local interactions at the interface. The figure was prepared using UCSF ChimeraX^32^.

In Patients 1–8, we identified the recurrent *FGF12* variant NM_004113.6:c.155 G > A (p.Arg52His), previously reported as a pathogenic gain-of-function change that enhances modulation of Nav channels (rs886039903). This single-nucleotide variant affects both FGF12 isoforms and can also be described as NM_021032.5:c.341 G > A (p.Arg114His) for the FGF12-A isoform (NM_021032.5). The substituted arginine resides within the highly conserved β4–β5 loop of the β-trefoil domain, which interacts with the C-termini of voltage-gated sodium channels (Fig. 3). As structural characterization of human FGF12–Nav complexes is limited to the X-ray–resolved interaction with the Nav1.5 C-terminal domain (PDB: 4JQ0), with no experimentally determined structures for neuronal isoforms such as Nav1.2 or Nav1.6, leaving current models for these complexes dependent on docking or homology-based predictions with inherent uncertainty. The position is invariant across human paralogs and vertebrate orthologs. Multiple in silico prediction tools (PolyPhen-2, SIFT, MutationTaster) indicate a deleterious effect, and conservation metrics further support functional constraint (phyloP100: 7.568). The variant is absent from large population datasets (gnomAD, 1000 Genomes, ExAC) and exhibits a recurrent de novo pattern across unrelated families, consistent with pathogenicity.

Functionally, Siekierska et al.^19^ demonstrated a paradoxical gain-of-function effect in which substitutions at this site - including the Arg-to-His change—disrupt specific FGF12-Nav interactions yet enhance the FGF12-mediated depolarizing shift of Na_v_ inactivation. In heterologous recordings with Na_v_1.6, both mutant isoforms produced significant depolarizing shifts in steady-state fast inactivation compared with wild type (FGF12-A: V₁/₂ − 56.4 ± 0.9 mV vs −63.4 ± 1.6 mV; FGF12-B: V₁/₂ − 65.8 ± 1.3 mV vs −74.9 ± 1.3 mV), predicting increased channel availability at subthreshold voltages and consequent neuronal hyperexcitability.

Patient 9 carries the NM_004113.6:c.148 G > A (p.Gly50Ser) variant, which has been reported multiple times in ClinVar in individuals with disorders associated with DEE. This variant is absent from population databases, including gnomAD. Gly50 lies directly within the interaction interface between FGF12 and Na_v_1.6/Na_v_1.2. Functional analysis by Seiffert et al. demonstrated a gain-of-function effect when the variant was co-expressed with Nav1.6: co-expression of Na_v_1.6 with the G50S mutant resulted in a statistically significant increase in current density compared with wild-type FGF12^7^. The similar effects on the voltage dependence of fast inactivation, together with the physical proximity of Gly50 to the Arg52His pathogenic hotspot, suggest that both mutated FGF12 proteins may interfere with channel function through a shared or a closely related mechanism.

Patients 10 and 11 carry a novel, maternally inherited *FGF12* missense variant, NM_004113.6:c.458 A > G (p.Glu153Gly), which affects both protein isoforms and lies within the intrinsically disordered region. The affected glutamate residue shows strong evolutionary conservation (phyloP100: 7.636). Pathogenicity predictions are conflicting: PolyPhen-2 suggests a benign effect, whereas SIFT and MutationTaster indicate deleterious consequences.

Functional evidence supports the importance of this region: FGF12 acts as a critical bridging protein within nucleolar NOLC1/TCOF1 complexes essential for ribosome biogenesis, and deletion of residues 131–181 abolishes binding to both NOLC1 and TCOF1. Because the Glu153Gly substitution occurs within this key interaction region, it may impair assembly of these nucleolar complexes, leading to defective RNA polymerase I regulation and subsequent ribosome biogenesis dysfunction. Of note, a different substitution at the same codon (p.Glu153Lys) has been reported in ClinVar as a variant of uncertain significance in an infant with recurrent seizures beginning at three months of age, further supporting the functional relevance of this residue.

Disruption of NOLC1/TCOF1–FGF12 assemblies is expected to hinder proper engagement of RNA polymerase I with nucleolar processing machinery, triggering nucleolar stress and activation of p53-mediated apoptotic pathways in neurons. Together, these mechanistic insights suggest that the Glu153Gly variant may represent a pathogenic mutation that causes epilepsy through a ribosomal dysfunction mechanism distinct from the classical sodium channel–mediated effects of other FGF12 mutations.

In Patient 12 we identified a 33-kb intragenic amplification (hg38:chr3:192,314,700 - 192,347,822) with predicted breakpoints by chromosomal microarray analysis within introns 3 and 4 of the MANE Select transcript (i.e., encompassing a single out-of-frame exon present in all coding *FGF12* isoforms). Exon-level tandem duplications within coding sequence are most plausibly disruptive, as they frequently introduce frameshifts or aberrant splice junctions that trigger nonsense-mediated decay (NMD). Even if an in-frame duplication were retained, exon 4 encodes part of the conserved β-trefoil core that forms the FHF–Na_V_ interaction module; internal duplication of this tightly packed element is expected to impair folding, destabilize the protein, and promote proteasomal degradation rather than produce a stable product.

### *FGF12* landscape

Through a PubMed search for *FGF12* epilepsy, FGF-12 epilepsy, FHF1 epilepsy, FHF-1 epilepsy (last search: November 2025), we identified 25 papers, including 49 patients (Supplementary Table). 1 patient has autism spectrum disorder but not epilepsy. 27 patients had the identical recurrent monoallelic p.Arg52His (c.155 G > A) variant, 11 had the monoallelic p.Gly50Ser (c.148 G > A) variant, with 2 other variants reported in individual patients, one of which was biallelic. 9 have chromosomal duplications affecting the *FGF12* gene.

Seizure onset was in the first 4 months of life in all patients, except those with chromosomal duplications, 8/9 of whom presented aged 12 months – 4 years. 31/49 had moderate to severe ID and 18/49 had ASD or suggestive features. 31/49 report partial or full responsiveness to SCB and 6 responded to sodium valproate. Lag time to SCB treatment is largely not reported except in a recent multicenter French series^11^. We note that outcomes are far better in the recent French series than were previously described, and that these patients largely received prompt SCB therapy, which is not reported in the early literature. Hypsarrhythmia was reported in 4 patients, two had chromosomal duplications, one with a biallelic c.259 G > A variant, and one with the recurrent p.Arg52His (c.155 G > A)^12,20,21^. Epilepsy was largely described as focal, or focal and generalized combined. MRI brain was normal or with non-specific signs in most but 5 had cerebellar atrophy. No other systemic features were reported except calcinosis cutis in a single patient with the recurrent p.Arg52His (c.155 G > A) and dysmorphism in a patient with a second duplication. There is one striking report of a patient with the recurrent p.Gly50Ser (c.148 G > A) variant developing syncope, and ictal asystole requiring pacemaker implantation at the age of 7 years. However, there are no other reports of cardiac arrythmias^22^.

### Integrated clinical phenotype and treatment response across reported FGF12-related epilepsy cases

**Fig. 4:**
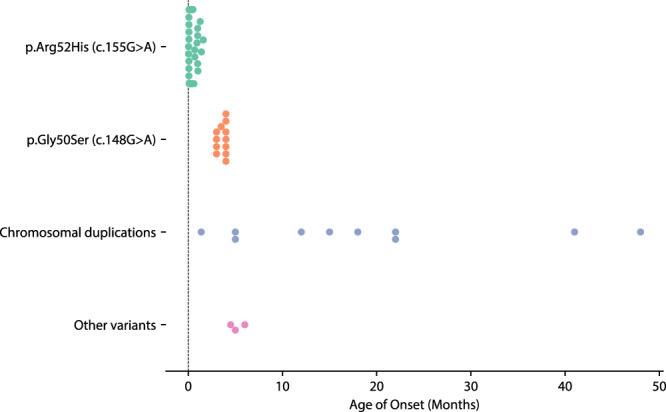
Clustering of age of seizure onset according to variants/chromosomal duplications in previously reported and newly described patients. Other variants refers to p.Glu153Gly (c.458 A > G) in Patients 10 and 11, and p.Glu87Lys (c.259 G > A) biallelic variant reported by Ohori et al. Age of seizure onset is based on reports in the literature summarized in the Supplementary Table.

Onset in both our cohort and previously reported cases occurred predominantly in the neonatal period or early infancy among individuals with single-nucleotide variants. The only exception in our series was a patient with a chromosomal duplication who presented at 22 months, consistent with the later onset described in other patients with chromosomal abnormalities. As illustrated in Fig. 4, the variants identified in our cohort cluster with those previously reported in the literature.

Seizure semiology and electroencephalographic features were broadly concordant between our patients and earlier reports. Most individuals exhibited focal-onset seizures, although several were described as having combined generalized and focal epilepsy, and a minority had exclusively generalized seizures. Multifocal interictal EEG abnormalities were observed in both our cohort and prior cases, although some patients in each group demonstrated normal interictal recordings. None of our patients exhibited hypsarrhythmia, whereas this pattern was documented in four previously reported individuals, including one carrying the recurrent p.Arg52His variant.

Treatment responses also showed both overlap and divergence. In the published literature, 31 of 49 patients demonstrated at least partial improvement with sodium channel–blocking agents, whereas eight patients in our cohort achieved either seizure freedom or a marked reduction in seizure frequency. Developmental outcomes in four of our patients paralleled the majority of previously described cases, with moderate to severe developmental delay or intellectual disability reported in 31 of 49 individuals. Notably, however, eight patients in our cohort had normal or near-normal developmental trajectories.

Neuroimaging findings were similarly consistent across cohorts. Most patients had normal MRI studies, although cerebellar atrophy was reported in five of 49 previously described individuals and was also identified in one patient in our series. We additionally observed ischemic changes in one patient as a sequela of status epilepticus, a finding not previously documented.

## Discussion

We describe 12 patients with *FGF12*-related epilepsy, 8 of whom have the recurrent p.Arg52His (c.155 G > A) variant. As with previous patients reported, those with this variant presented with neonatal-onset seizures which were refractory to first-line ASMs (generally levetiracetam) and not consistent with any ILAE epilepsy syndrome. Among these patients, 6 infants were started on SCBs within 2 months of seizure onset, with resolution of seizures or significant seizure frequency reduction and later entirely normal development; 2 were seizure-free with mild developmental impairment/high functioning ASD. A further patient responded well to SCB with normal development until age 1 year, with deterioration following seemingly unrelated NMDA encephalitis (with positive antibodies), with 3 other patients, all with early status epilepticus, with poor developmental outcomes.

Improved outcomes appeared to be consistent with a range of SCBs, including those modulating fast inactivation such as oxcarbazepine, phenytoin, carbamazepine and lamotrigine, as well as lacosamide which enhances slow inactivation. We note that cenobamate was not administered to any patients in this series. Additionally, topiramate was effective in 4 patients which may relate to its putative secondary mechanism of action reducing the frequency of activation of voltage-sensitive sodium channels^23^. Perampanel was helpful in one patient, with no clear mechanistic explanation, yet is often empirically used in rare genetic epilepsies with good efficacy^24^. The lack of efficacy of levetiracetam is consistent with what has been reported in *SCN8A*-related epilepsy, where it is often unhelpful and can at times worsen seizures^25,26^.

*FGF12*-related epilepsy has been previously categorized in association with DEEs (DEE47), and indeed almost all patients reported prior to a recent multicenter study had poor neurodevelopmental outcomes, for example, in the initial cohort described by Trivisano et al.^10^. Conversely, Pierret et al. describe 7 patients out of 10 having normal neurodevelopmental outcomes, with 3 having mild intellectual disability, yet even these patients could not be described as having DEEs.^11^. Most of our cohort is largely consistent with the French series, both in terms of epilepsy onset, pharmacoresponsiveness, and excellent outcomes.

We demonstrate a genotype-phenotype correlation regarding age of seizure onset both in our cohort and previously described patients, with those with the recurrent p.Arg52His (c.155 G > A) variant presenting in the first 2 months of life, while patients with p.Gly50Ser (c.148 G > A) presenting at age 3–4 months. Patients with chromosomal abnormalities typically presented much later, up to the age of 4 years, often with developmental regression associated with seizure onset.

However, in distinction with other channelopathies, such as *SCN1A* and *SCN2A*-related epilepsies with robust genotype-phenotypic patterns^27,28^, we found significant clinical heterogeneity in the neurodevelopmental outcomes of patients within the same recurrent variants, which could be explained by a number of factors. First, we propose that early SCB administration may modulate outcomes. We note that there is no indication that patients in the earlier publications were treated promptly with SCBs (for example those reported by Trivisano et al.^10^), whereas in our cohort and the French cohort, patients were diagnosed more promptly with ASMs transitioned to SCBs. Second, we acknowledge that with increasing availability of NGS panels, exome and more recently genome sequencing, patients with milder phenotypes are increasingly identified, and the patients we report may reflect the milder end of the phenotypic spectrum, while in previous decades similar patients were less likely to undergo genetic testing. Third, complex genetic or environmental factors may modify outcomes and penetrance may affect the previously characterized gain-of-function effect of the recurrent variants described^19^.

*FGF12* interacts with epilepsy-associated sodium channels Na_v_1.2 with a loss-of-function effect on voltage-dependent fast inactivation, and a gain-of-function effect on Na_v_1.6^19^ with an associated effect on time constants of fast inactivation of both channels and slow inactivation of Na_v_1.6 particularly in missense variants, however loss of function effects can be dominant in duplications^7^. SCBs appear to enhance the channels’ fast inactivation with more hyperpolarized potentials. Paralleling well-characterized *SCN2A* and *SCN8A* disease-causing variants, phenotypic variability and response to SCBs between *FGF12* variants may be related to subtle gain vs. loss of function effects. While the heterogeneity we observed within the same recurrent variant in our cohort cannot be explained by this possibility, more subtle structural variants may play a role. Two recent publications report on patients with DEEs identified to have structural variants in *FGF12* on long-read sequencing leading to structural disruption, with a shift in inactivation to hyperpolarized potential, hypothesized to lead to a reduction in FGF12 expression and therefore loss of function effects^16,20^. Additional structural variants not identified on routine genetic testing may therefore modulate downstream effects, including in recurrent variants leading to clinical variability.

Neuroimaging in our cohort was similar to published literature with most patients having no structural abnormality, and 1 patient demonstrating cerebellar atrophy as previously described and one with ischemic changes, likely secondary to status epilepticus^29^. EEG was non-specific, with both focal or multifocal/generalized changes, with one infant with a migratory ictal pattern. We note that many patients in the literature are reported to have combined focal and generalized epilepsy, yet seizure semiology and EEG findings of our patients suggest focal onset including where generalized tonic-clonic seizures were also reported.

Sudden unexpected death in epilepsy with cardiac arrhythmia have been reported in a mouse model of the recurrent p.Arg52His (c.155 G > A) variant^30^. One case report describes a child with syncope, and ictal asystole requiring pacemaker implantation at the age of 7 years with Patient 9’s p.Gly50Ser (c.148 G > A) variant^22^. Although our patients underwent normal cardiology work-up, animal data together with this report emphasize the need for cardiac surveillance in patients with *FGF12*-associated epilepsy.

In conclusion, the expanded *FGF12*-related epilepsy phenotypic spectrum we demonstrate here may reflect improvement of epilepsy and developmental outcomes as a result of prompt SCB therapy. This emphasizes the role of early genetic testing and precision therapy on developmental and epilepsy outcomes. Prospective, long-term evaluation of individuals with *FGF12*-related epilepsy will be valuable in determining their long-term seizure and developmental outcomes.

## Methods

### Study cohort—deep phenotyping and genetic work-up

Individuals with variants or duplications in *FGF12* were identified via international professional networks. Data collected from pediatric neurology departments from 5 tertiary pediatric epilepsy centers in the UK, Canada, Israel and USA included demographic information, family history, seizure semiology and characteristics (including age of onset, efficiacy of ASMs), EEG findings, MRI findings, and neurodevelopmental outcomes.

1 diagnosis was made on chromosomal microarray, 5 on genome sequencing, 5 on exome sequencing and one on a Next Generation Sequencing panel. Variants were classified according to American College of Medical Genetics and Genomics (ACMG)/ Association for Molecular Pathology (AMP) criteria.

Parents or caregivers of all individuals described in the manuscript gave written consent to participate and publish clinical details including MRI images and EEGs. Approval was given by Institutional Review Boards in each participating institution according to local protocol (UCL Great Ormond Street Institute of Child Health [IRAS reference 13/LO/0171], Sheba Medical Center [SMC-7786-10], SickKids Hospital [SickKids Research Ethics Board 1000077695], University of Michigan [IRB no. HUM00284259], Boston Children’s Hospital [BCH Institutional Review Board X10-04-0197].

### In silico pathogenicity prediction and conservation metrics

Pathogenicity predictions were obtained from PolyPhen-2, SIFT, and MutationTaster using default settings. Evolutionary conservation was assessed using phyloP100 vertebrate scores retrieved from the UCSC Genome Browser. Paralog and ortholog conservation of affected residues was evaluated using multispecies alignments generated with Clustal Omega.

### Structural modeling and visualization

Structural analyses of missense variants were performed using experimentally determined FGF12–Nav channel complexes. Specifically, structural visualization employed the crystal structure of the FGF12–Nav1.5–calmodulin complex (PDB 4JQ0; Wang et al.)^31^. All renderings, distance measurements, and side-chain mutagenesis (e.g., Arg52His, Glu153Gly, Gly50Ser) were performed in UCSF ChimeraX^32^. Mutant side-chain conformations were modeled using the Dunbrack rotamer library. Interatomic distances, hydrogen bond geometry, and van der Waals contacts were analyzed using the ChimeraX “contacts,” “hbonds,” and “distance” tools. Changes in residue orientation and formation of new interaction patches were evaluated qualitatively and by manual inspection.

### Protein-protein interaction annotation

Structural regions implicated in binding to voltage-gated sodium channels were annotated based on published biochemical and crystallographic data. Interaction interfaces between FGF12 and Nav1.2/Nav1.6 C-terminal domains were assigned according to Siekierska et al. (2016), Wang et al. (2014), and Seiffert et al. (2022)^7,19,31^. For the Glu153Gly analysis, the functional domain involved in nucleolar NOLC1/TCOF1 interactions was defined using previously reported FGF12 deletion constructs, specifically the 131–181 region required for binding.

## Supplementary information

Supplementary Table 1

## Data Availability

This study is a clinical case series involving individuals with rare genetic conditions. Owing to the small number of participants and the risk of re-identification, the individual-level data cannot be made publicly available. In order to promote appropriate data sharing while safeguarding participant confidentiality, all clinically relevant genetic variants identified in this study will be submitted to ClinVar, together with relevant phenotypic information, in line with participant consent and recognized best practice. This will ensure that variant-level data are accessible to the wider research and clinical community. Requests for access to additional aggregated or anonymised data may be considered by the corresponding author, subject to approval through institutional governance processes and in compliance with UK data protection legislation.
