# Supplementary material for: Expanding the phenotypic spectrum of FGF12-epilepsy—does prompt precision therapy affect outcomes?
Source: NPJ Genom Med. 2026 May 25;11:41. doi: 10.1038/s41525-026-00581-0 (PMC13385693; doi:10.1038/s41525-026-00581-0)
Supplement: Supplementary file 1 — Supplementary Table 1 [file 41525_2026_581_MOESM1_ESM.pdf]

PATIENT ID	Reference	Gender/Age at last observation	FGF12 variant, inheritance	Epilepsy onset	Seizure type	Epilepsy Type	SE (frequency)	Interictal EEG	Ictal EEG	ASM	ASM efficacy	ID	ASD and other disturbances	First brain MR/age	Second brain MR/age	Lag between seizure onset and SCB
1	Siekierska et al. 2016	F/died age 7y (SE)	p.Arg52His (c.155G>A)* presumed gonadal mosaicism	14d	TS	Combined generalized and focal epilepsy	frequent	Slow BG, multifocal SW (onset)	Generalized onset (tonic seizure)	PB, VPA, PHT, GVG, TPM, CZP; PN	Resistant to ASMs; best response to PHT	severe	stereotypies, absent eye contact, acquired microcephaly	Normal/6m	Cerebellar atrophy/6y	Not reported
2	Siekierska et al. 2016	M/died age 3y6m (unknow cause)	p.Arg52His (c.155G>A)* presumed gonadal mosaicism	28d	TS	Combined generalized and focal epilepsy	infrequent	Slow BG, multifocal SW (onset)	Generalized onset (tonic seizure)	PB, VPA, PHT, GVG, TPM	Resistant to ASMs; best response to PHT	severe	stereotypies, absent eye contact, acquired microcephaly	Normal/4m	Cerebellar atrophy/3y	Not reported
3	Al-mehmadi et al. 2016	M/3y	p.Arg52His (c.155G>A) de novo	2d	FTS, FBTCS	Focal epilepsy	frequent	Slow BG, multifocal SW	Focal to bilateral t-c	LEV, PB, KD	Resistant to ASMs	severe	n.a.	Normal/5d	Cerebral atrophy/2y	Not reported
4	Al-mehmadi et al. 2016	F/16y	p.Arg52His (c.155G>A) de novo	42d	FTS, MS, FBTCS	Combined generalized and focal epilepsy	frequent	Slow BG, multifocal SW	n.a.	PHT, PER, VNS	Resistant to ASMs; partially responsive to PHT and VNS	severe	n.a.	Normal/1y	Cerebellar atrophy/8y	Not reported
5	Al-mehmadi et al. 2016	F/8y	p.Arg52His (c.155G>A) de novo	2d	FTS, FS	Focal epilepsy	frequent	Slow BG, multifocal SW	n.a.	PHT, PRG, PER, VNS	Resistant to ASMs	moderate	yes	Normal/21d	Bilateral mesial temporal sclerosis (R>L), mild prominence of cerebellar folia/12y	Not reported
6	Guella et al. 2016	F/3y3m	p.Arg52His (c.155G>A) de novo	2d	FTS	Focal epilepsy	no	Discontinuous, multifocal SW (onset); increase of diffuse/multifocal SW; from 10m normal EEG (FU)	Generalized onset	PB, LEV, TPM, PHT, CBZ	Responsive to PHT and CBZ	no	no	Normal/3d	No	17d
7	Guella I. et al. 2016	F/15y	p.Arg52His (c.155G>A) de novo	2d	FS, FBTCS	Focal epilepsy	n.a.	Slow BG left temporal SW (onset); slow BG and multifocal SW (FU)	Generalized onset (tonic seizure)	PB, TPM, LTG, RUF	Responsive: to RUF and LTG,	moderate	yes	Mild Chiari I /14d	Mild Chiari I /2y	Not reported
8	Villeneuve et al. 2017	M/9y	p.Arg52His (c.155G>A) de novo	1d	AS, FS	Combined generalized and focal epilepsy	n.a.	Normal BG, multifocal spikes (onset); focal spikes (FU)	Generalized onset (tonic seizure)	PB, GVG, CBZ, CLB, ESM, KD	Partially responsive to CBZ, later responded to fluoxetine	mild	very tight	Tight T2 weighted hyper intensity of the parietal region, cerebellum and brain stem/15d	n.a.	Not reported
9	Shi RM. et al. 2017	M/15y1m	arr[hg19] 3q28q29 ×1, 0.58-Mb gain, including FHF1 gene, *de novo*	3y5m	GTCS, FS, FTS	Combined generalized and focal epilepsy	no	Slow BG, Frontal SW (onset); Slow BG, multifocal SW (FU)	Generalized onset (AS, TS, GTCS) Focal seizures: R or L hemisphere	PB, CLB, VPA, KBr, PHT, LEV, NZP	Best response to high dose of PHT	severe	yes, stereotypies, absent eye contact	Mild cerebral and cerebellar atrophy/3y	Mild cerebral and cerebellar atrophy/8y	2 years
10	Takeguchi R. et al. 2018	M/33y3m	p.Arg52His (c.155G>A)^, inherited from unaffected mother with mosaicism	7d	TS, ES	Focal epilepsy	monthly	Suppression burst (onset); slow BG with focal spikes (FU)	n.a.	VPA, PB, PHT, CZP, AZA, PHT, GBP	Partially responsive to PHT, CZP and VPA	severe	yes, stereotypies, absent eye contact	Mild enlargement of lateral ventricle/7y	Mild enlargement of lateral ventricle/13y	Not reported
11	Takeguchi R. et al. 2018	M/2y6m	p.Arg52His (c.155G>A) de novo	1d	FTS, FS, FBTCS	Focal epilepsy	no	Slow BG, multifocal and diffuse SW (onset); slower BG multifocal SW (FU)	Generalized onset. Seizure activity migrated from one region to another	PB, PN, CBZ, CLB, VPA, ZNS, LEV, KBr, PHT	Best response to PHT and high-dose of PB	severe	No, poor eye contact, congenital microcephaly	Mild cerebral atrophy/6m	Diffuse cerebral atrophy/1y7m	Not reported

**12**	Paprocka et al. 2019	M/4y6m	p.Gly50Ser (c.148G>A) de novo	4m	TS, MS, ES, FTS,FBTCS	Combined generalized and focal epilepsy	no	Normal (onset); generalized and focal paroxysmal in temporal regions (FU)	Generalized polyspikes, spike-SW complexes	PB, CBZ, VPA, LEV, GVG, steroids, PHT	Best response to PB and PHT	moderate	yes	Normal/4m	No	Not reported
**13**	Epilepsy Genetics Initiative, 2019, published by Trivisano et al. 2020	M/5y8m	p.Arg52His (c.155G>A) de novo	31d	TS	Combined generalized and focal epilepsy	yes (twice)	Normal BG, multifocal SW (onset); slow BG and multifocal SW (FU)	Diffuse onset	PB, PHT, CBZ, LEV, RTG, VPA	Partially responsive to VPA	moderate	yes, stereotypies, absent eye contact	Mild cerebral atrophy/4m	Mild cerebral atrophy/2y9m	Not reported
**14**	Trivisano et al. 2020	F/1 m	p.Arg52His (c.155G>A) de novo	2d	FS	Focal epilepsy	no	Discontinuous and multifocal SW (onset); slow BG with increase of diffuse/multifocal SW	n.a.	PB, LEV, PN/PLP, TPM, CBZ, PHT	Resistant to ASMs	moderate	No, rapid mood swings, congenital microcephaly	Normal/5d	Normal/10d	Not reported
**15**	Trivisano et al. 2020	F/13y	p.Arg52His (c.155G>A) de novo	3d	A, FTS, FBTCS	Combined generalized and focal epilepsy	frequent	Slow BG, multifocal SW (onset); Slow BG and multifocal and diffuse SW (FU)	L hemisphere; R frontal/frontotemporal	PB, LEV, PHT, OXC, LCM, CLZ, PER, TPM, VNS, KD	Resistant to ASMs	moderate	yes, severe obsessive behaviour	Normal/1y	Normal/4y	Not reported
**16**	Trivisano et al. 2020	M/2y10m	p.Arg52His (c.155G>A) inherited from affected mother (mosaicism)	8d	FS, TS, FBTCS	Unknown	yes (twice)	Normal BG multifocal SW (onset) Slow BG and posterior SW (FU)	R central	VPA, CZP, LEV, GVG, CBZ, PN, PLP, PB, Folic Acid, Biotine	ASMs-responsive	moderate	yes, stereotypies, absent eye contact	Normal/21d	Normal/2y	Not reported
**17**	Trivisano et al. 2020	M/4y2m	p.Gly50Ser (c.148G>A) inherited from affected father (mosaicism)	4m	TS, FBTCS	Unknown	no	Normal	n.a.	LEV, VPA, LTG	ASMs-responsive	no	no	Normal/4m	Normal/3y4m	Not reported
**18**	Verheyen et al 2020	F/7y	3q28q29 duplication (191874052_192456936x3) de novo	4y	GTCS, TS	Generalized epilepsy	No	Primary generalized and bioccipital epilePHTic discharges	Background suppression with periodic bursts of slow wave activity during nonconvulsive status epilePHTicus	VPA, PHT, CBZ, LTG, LEV, TPM, CLB	Partially responsive to PHT. Responsive to LTG, LEV, TPM, CLB	Severe	No	Normal/7yr	No	Not reported
**19**	Willemsen et al 2020	M/10Y	191 860 089-192 451 114	12m	GTCS, atonic, tonic, myoclonic, autonomic	Combined generalized and focal epilepsy	Frequent	slowed background activity and multifocal epilePHTic discharges	Not reported	VPA, CBZ, LTG, LEV	Resistant to ASMs	Severe	Not reported	Bilateral delayed myelination in the parieto occipital region	No	Not reported
**20**	Willemsen et al 2020	M/4Y	191 876 968-192 454 685	15m	GTCS, myoclonic	Generalized	Frequent	Generalized epilePHTic activity	Not reported	VPA	Resistant to ASMs	Moderate	ASD level 3	Mild prominence of the subarachnoid space in the frontal regions bilaterally	No	Not reported
**21**	Rochtus et al. 2020	M/4Y	p.Gly50Ser (c.148G>A)	4m	Generalized	Combined generalized and focal epilepsy	Frequent	Right frontotemporal focus on EEG	Not reported	Not reported	Not reported	Present	Global developmental delay, methylmalonic acidemia	Normal/6m	Normal/11m	Not reported

**22**	Tian et al 2021	M/2Y	p.Arg52His (c.155G>A) de novo	15d	GTCS, MS	Combined generalized and focal epilepsy	Frequent	Burst-suppression, later central parietal spikes	Not reported	TPM, VPA	Responsive to VPA, TPM combination	Severe	No eye contact	Normal	No	n/a
**23**	Tian et al 2021	F/3Y	p.Arg52His (c.155G>A) de novo	18d	GTCS	Combined generalized and focal epilepsy	Frequent	Burst-suppression, later multifocal slow SW	Not reported	LEV, TPM, VPA	Responsive to VPA, TPM combination	Severe	Severe motor impairment	Normal	No	n/a
**24**	Tian et al 2021	M/not reported	p.Arg52His (c.155G>A) de novo	1m	ES	Combined generalized and focal epilepsy	Frequent	Hypsarrythmia	Not reported	PB, LEV, TPM, VPA	Responsive to VPA, TPM combination	No	No	Not reported	No	2m
**25**	Oda et al. 2019	F/14Y	3q28-q29(3qter) duplication, 9p24.3(9PHTer)-p22.3 deletion, (14.2 Mb), 9p22.3-p21.1 duplication (17.4 Mb)	6w	Behavioral arrest, TS, ES	Combined generalized and focal epilepsy	Frequent	Hypsarrythmia aged 11 months	Not reported	Not reported	Not reported	Severe	Dysmorphism, low weight and height	Normal/41d	Mild frontal lobe atrophy/4y	Not reported
**26**	Kim et al. 2020	M/9YR	p.Arg52His (c.155G>A) de novo	3d	CS, GTCS	Focal epilepsy	Frequent	Burst-suppression, multifocal sharp waves	Not reported	PB, LEV, CLB, CZP, PHT	Partially responsive to PHT	Severe	No speech	Normal	Not reported	2y
**27**	Kim et al. 2021	Not reported	p.Arg52His (c.155G>A) de novo	7d	Not reported	Not reported	Not reported	Not reported	Not reported	Not reported	Not reported	Severe	Not reported	Not reported	Not reported	Not reported
**28**	Seiffert et al 2022	M/11Y	p.Gly50Ser (c.148G>A) de novo	3m	GTCS, A	Combined generalized and focal epilepsy	Frequent	Multifocal bihemispheric epilePHTic activity	Not reported	OXC, VPA	Partial	Moderate	ASD	Not reported	No	Not reported
**29**	Seiffert et al 2022	M/4.5Y	p.Ser8Phe (c.23C>T) according to NM_021032.4	NIL	n/a	n/a	Nil	Normal	Nil	n/a	n/a	Present	ASD	Normal	Normal	n/a
**30**	Marin-Hernandez et al 2020	M/5m	p.Arg52His (c.155G>A)	7d	Not reported	Not reported	frequent	Normal	Not reported	PHT, LEV	PHT	Not reported	Calcinosis cutis	Abnormal signal in thalamus and basal ganglia/5m	No	Not reported
**31**	Straka et al. 2023	F/not reported	p.Arg52His (c.155G>A) de novo	Neonatal	Focal	Focal epilepsy	frequent	Normal	Not reported	Not reported	Not reported	Severe	Not reported	Normal	Not reported	Not reported
**32**	Ohori et al. 2023	F/8.5Y	62-kb partial deletion of exon 6, 3' untranslated region and 169-kb intragenic-tandem duplication exons 3 and 4	5m	Epileptic spasms	IESS	Aged 8m and later during febrile illnesses	Hypsarrythmia aged 5m	Not reported	ACTH, VPA, ZNS, PHT, PB, LEV, CLB	VPA, ZNS, PHT, PB	Severe	Not reported	Normal/7m	Cerebral atrophy/1.5y	3m
**33**	Ohori et al. 2023	M/11Y	p.Glu87Lys (c.259G>A) biallelic	5m	Epileptic spasms	IESS	Frequent	Hypsarrythmia	Not reported	VPA, TPM, VGB, PB, CBZ, CBD	CBZ partial improvement	Severe	Not reported	Mild cerebral atrophy/1y	Generalized atrophy, white matter changes/6y	Not reported
**34**	Saleem et al. 2024	F/3Y	p.Arg52His (c.155G>A) de novo	3w	TS	Focal epilepsy	Frequent	Centrotemporal discharges	Not reported	LEV, PHT, CLB, PB, OXC, LTG, LAC	PHT, TPM	Mild speech delay	No	Normal/3w	No	Immediate
**35**	Abraham et al. 2024	F/22Y	3q28 duplication:19187 3173-192 456 745 (x3) de novo	22m	GTCS	Not reported	4 times, died aged 22y following status	Normal	Not reported	PHT, multiple ASMs not detailed	PHT only	Severe	Not reported	Normal/22m	Prominent perivascular spaces in midbrain and periventricular white matter/18y	6m

**36**	Fauqueux et al. 2025	M/6.5Y	3q28q29 duplication:192 18 2 695_192 719 315 (×3)	18m	GTCS	Focal and generalized epilepsy	monthly	Generalized delta rhythm, triphasic waves, multifocal spikes	Bilateral tonic-clonic seizure with suppression following.	LEV, VPA, LTG, CZP, CLB, TPM, PB, CBZ, PHT, FLB	CBZ, PHT, PB, FLB	Severe	Regresson on seizure onset	Normal/18m	Mild cerebal and cerebllar atrophy/4y	4y
**37**	Fauqueux et al. 2025	M/7Y	3q28q29 duplication:192 18 2 695_192 719 315 (×3)	5m	Focal clonic movements	Focal epilepsy	2 months SRSE aged 7 y	Delta encephalopathy, bilatearl frontal spike-wave discharges	Not reported	VPA< LEV, OXC, LTG, TPM, LAC, CZP, PHT, PER, CBD, CLB	PHT, ZNS when in SRSE	Severe	Regression aged 15m	Normal/6m	Normal/2y	Not reported
**38**	Piotrowski et al. 2025	M/9Y	p.Gly50Ser (c.148G>A) de novo	4m	TS	Focal epilepsy	4 months on episode	Normal	Not reported	CZP, PHT, VPA, ZNS, LEV	VPA, ZNS - unclear	Mild	Autistic traits. Syncope and ictal asystole	Normal/4m	Not reported	No regular SCB
**39**	Sunnetci-Akkoyunlu et al. 2025	F/6Y	p.Arg52His (c.155G>A) de novo	Not reported	Not reported	Focal epilepsy	Not reported	Disorganized background	Not reported	Not reported	Not reported	Not reported	Not reported	Thin corpus callosum, decreased white matter volume	Not reported	Not reported
**40**	Pierret et al. 2025	M/4Y	p.Arg52His (c.155G>A) likely de novo	3d	TS, GTCS	Focal and generalized epilepsy	Rare	Normal	Not reported	LEV, CBZ, PHT	CBZ, PHT	No	No	Normal/1m	Not reported	7w
**41**	Pierret et al. 2025	F/22Y	p.Arg52His (c.155G>A) de novo	30d	TS, "focal seizures"	Focal and generalized epilepsy	Rare -2 episodes 18y	Normal	Left spikes and fast activity with contralateral diffusion	PB, VGB, PHT, CBZ, LEV, VPA, PER, BRV, LTG, LAC	CBZ, PHT, LTG partial	Mild	No	L. hippocampal atrophy/8y	Bilateral polymicrogyria /17y	Not reported
**42**	Pierret et al. 2025	F/2Y	p.Arg52His (c.155G>A) de novo	38d	TS, cyanosis	Combined generalized and focal epilepsy	Rare, responsive to PHT	Right frontal spikes	Diffuse flattening, slowing	CBZ, PHT, VPA, CLB, VGB, LEV	CBZ partial, PHT yes	Mild	Yes	Normal/2m	Not reported	1.5m
**43**	Pierret et al. 2025	F/2Y	p.Arg52His (c.155G>A) de novo	48d	Focal, GTCS	Combined generalized and focal epilepsy	No	Right posterior central spike waves	Not reported	CBZ	CBZ	No	No	Normal/3m	Not reported	1m
**44**	Pierret et al. 2025	F/38Y	p.Gly50Ser (c.148G>A) de novo	3m	GTCS	Generalized epilepsy	No	Usually normal. Post-ictal - right emporo-occipital spike wave	Not reported	PHT, LTG	PHT, LTG	No	No	Normal/23y	Not reported	1m
**45**	Pierret et al. 2025	M/4Y	p.Gly50Ser (c.148G>A) de novo	3m	TS, "focal seizures", absence	Combined generalized and focal epilepsy	No	Sleep: left frontal spike waves	Teft temporal rhythmic spike waves and spikes	CBZ	CBZ	No	No	Normal/3m	Not reported	2m
**46**	Pierret et al. 2025	F/8Y	p.Gly50Ser (c.148G>A) de novo	3m	TS, "focal seizures"	Combined generalized and focal epilepsy	At diagnosis responsive to PHT	Normal	Right temporal and bifrontal progression	CBZ, PHT	CBZ, PHT	No	No	Normal/3m	Normal 4y	2.5m
**47**	Pierret et al. 2025	F/6Y	p.Gly50Ser (c.148G>A) de novo	4m	TS, "focal seizures", generalized seizures	Combined generalized and focal epilepsy	At diagnosis responsive to PHT	Left frontotemporal spikes activation in sleep	Not reported	OXC, LEV, VGB	OXC	No	No	Normal/4m	Left temporal pole atrophy with subcortical T2 hypersignal/20 m	8m
**48**	Pierret et al. 2025	M/9Y	p.Gly50Ser (c.148G>A) de novo	4m	GTCS	Combined generalized and focal epilepsy	No	Normal	Bitemporal spikes	LTG, VPA, LEV	LTG	No	No	Normal/4m	Normal/3y	2.5y
**49**	Pierret et al. 2025	F/10Y	p.Gly50Ser (c.148G>A) de novo	4m	TS, "focal seizures", GTCS	Combined generalized and focal epilepsy	No	Left temporal spike waves	Not reported	VPA, TPM, PHT	PHT	Mild	No	Normal/4m	Normal/4y	Not reported

Supplementary Table 1: Phenotype and genotype data for previously reported patients with *FGF12* -epilepsy
